# Preoperative reference values for breast cancer patients using the BREAST-Q

**DOI:** 10.1016/j.breast.2024.103832

**Published:** 2024-10-31

**Authors:** Charlotta Kuhlefelt, Jussi P. Repo, Veera Rasi, Tuomo Meretoja, Tiina Jahkola, Susanna Kauhanen, Pauliina Homsy

**Affiliations:** a Division of Musculoskeletal and Plastic Surgery, Department of Plastic Surgery, University of Helsinki and Helsinki University Hospital, Helsinki, Finland; b Unit of Musculoskeletal Disease, Department of Orthopedics and Traumatology, Tampere University Hospital and University of Tampere, Tampere, Finland; c Division of Breast Surgery, Comprehensive Cancer Center, Helsinki University Hospital and University of Helsinki, Helsinki, Finland

**Keywords:** Breast cancer, Breast reconstruction, Patient-reported outcomes, Health-related quality of life, BREAST-Q

## Abstract

**Introduction:**

The BREAST-Q can be used to evaluate the health-related quality of life (HRQL) of breast cancer patients. Data interpretation is limited by the lack of previous reference values based solely on patients with a recent breast cancer diagnosis.

**Methods:**

A total of 627 patients, all with newly diagnosed breast cancer, were asked to participate in the study. The BREAST-Q modules for mastectomy and breast-conserving surgery were used. The results for the scales were reported as mean with standard deviation (SD). The effect of patient characteristics, including age, body mass index (BMI), and ASA-classification on the HRQL were analyzed with multiple linear regression.

**Results:**

In total, 315 patients (50.2 %) participated. The mean (SD) age was 60.3 (10.1) years. Mean scores (SD) were the following: Psychosocial Well-being 70.8 (15.0), Sexual Well-being 58.2 (15.1), Satisfaction with Breasts 59.9 (15.6), and Physical Well-being: Chest 81.7 (15.7). The psychosocial well-being, sexual well-being, and satisfaction with breasts were all similar compared to the normative mean scores of the scales. The physical well-being of the chest was lower than the normative mean value (p < 0.001). Psychosocial well-being (p = 0.007), sexual well-being (p = 0.007), and satisfaction with breasts (p < 0.001) were lower in patients with higher BMI. Younger patients reported lower physical well-being of the chest (p < 0.001).

**Conclusions:**

This study established preoperative reference values for the BREAST-Q in breast cancer patients. This data can be used to evaluate the HRQL in breast cancer patients accurately.

## Highlights

• This study provides preoperative HRQL reference values for breast cancer patients.
• The established reference values can be applied to several BREAST-Q modules.
• Breast cancer reduces the physical well-being of the chest.
• Accurate reference values improve the assessment and follow-up of cancer patients.

## 1 Introduction

Breast cancer has been shown to significantly decrease the health-related quality of life (HRQL) of patients [1,2]. Patient-reported outcome measures (PROMs) are becoming increasingly valued when assessing the effect of breast cancer treatment and surgical outcomes [3]. It is essential to have access to preoperative data to evaluate the impact of the treatments on the HRQL.

The BREAST-Q is a widely used, breast-specific PROM evaluating patients’ HRQL. It is designed for use on patients who have undergone breast surgery, and there are modules available for different surgical approaches [[4], [5], [6]]. The results of the BREAST-Q are often compared with normative mean scores for the questionnaire, developed from answers of a healthy control group without a prior history of cancer or breast surgery [7,8].

Although several studies have presented normative values for different BREAST-Q modules, these all include patients without breast cancer [[9], [10], [11]]. To our knowledge, no baseline HRQL scores have been published for patients newly diagnosed with breast cancer. Comparing the results of women with breast cancer or breast surgery to the population without any prior history of either breast cancer or breast surgery is suboptimal, as previous studies have shown a significant effect of cancer and surgery on the HRQL [[12], [13], [14]].

The aim with this study was to assess the preoperative HRQL of patients diagnosed with breast cancer using the BREAST-Q, and thus establish reference values for patients with a pre-existing breast cancer diagnosis. These baseline values will ease the interpretation of postoperative HRQL and aid in determining the effect of surgical treatment.

## 2 Methods

**Fig. 1:**
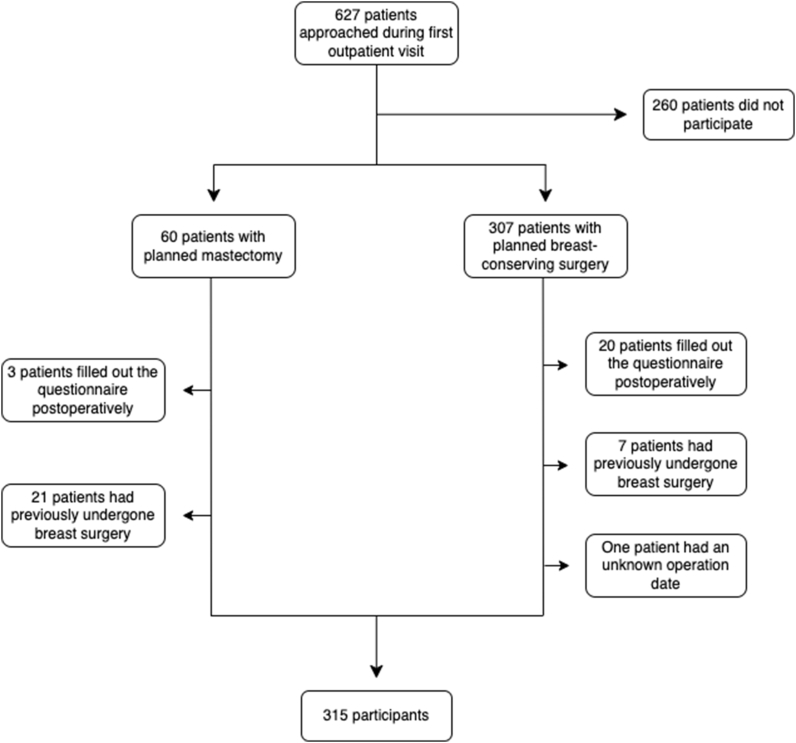
Flowchart showing the patient selection for the study.

A cohort study was performed on patients with a new diagnosis of breast cancer between December 2019 and March 2021 in Helsinki University Hospital Breast Cancer Unit. The patients were recruited during their first outpatient visit. Finnish-speaking women between the ages of 18 and 85 with recently diagnosed breast cancer, without prior breast surgery were included. Patients with previous breast cancer or breast surgery were excluded. Questionnaire forms that were filled out after the planned surgery were not included. The patients were given a physical questionnaire package including a background information form, information on the study, the BREAST-Q questionnaire, a consent form, and a pre-paid return envelope. The patients filled out the forms after the visit and mailed the filled-out questionnaire package to the Breast Cancer Unit. The answers were then transferred to electronic format using anonymized patient identification numbers. Patient selection process is shown in Fig. 1.

Information on general health status and underlying diseases was retrieved from the patients’ medical records. The study protocol was approved by the Helsinki University Hospital ethics committee (HUS/2737/2017). Written consent was obtained from all study participants.

### 2.1 Study questionnaire

The Finnish version of the BREAST-Q was used to assess the patients’ pre-operative HRQL [6]. The BREAST-Q modules for mastectomy and breast-conserving surgery were used, with the module chosen according to the planned surgery. The patients were asked to fill in four preoperative scales: Psychosocial Well-being, Sexual Well-being, Satisfaction with Breasts, and Physical Well-being: Chest [[4], [5], [6], [7]].

Psychosocial Well-being is assessed with ten questions addressing aspects of emotional health, social well-being, and body image. Sexual Well-being consists of six questions, assessing topics such as sexual confidence, comfort, and satisfaction with sex life. The Satisfaction with Breasts is assessed with four questions, focusing on clothing fit and appearance. The scale for Physical Well-Being:Chest consists of ten questions assessing symptoms in the chest area, including pain, discomfort, and tightness.

### 2.2 Statistical analysis

The BREAST-Q subscales were analyzed according to the developer's instructions [15]. If more than 50 % of the answers in a subscale were missing, the answers were excluded from further analysis. In cases with less than 50 % of the answers missing, the mean response from the other questions was used. The answers were rescaled using the nonlinear Rasch transformation method [16]. In each scale, 0 indicated the worst outcome and 100 the best outcome. Mean values and standard deviations (SD) were reported.

Multiple linear regression analyses were performed to evaluate the independent effects of age, body mass index, and ASA-classification on the HRQL. Collinearity was assessed using a variance inflation factor with a limit VIF <5. In all tests, p-values <0.05 were considered statistically significant. T-tests were used for comparison of mean values.

All statistical tests were conducted using IBM SPSS version 29 statistical software [17].

## 3 Results

A total of 627 patients were approached and given the questionnaires. Of these, 315 patients (50.2 %) participated in this study. None of the patients included in the study had any previous history of breast surgery, per study design, and only preoperatively filled BREAST-Q questionnaires were included. All patients had recently been diagnosed with breast cancer. The mean age was 60.3 years (SD 10.1). The mean time from the study questionnaire until surgery was 11 days (SD 15.1). The mean BMI of the patients was 26.7 (SD 4.8). Patient characteristics are presented in Table 1.

**Table 1:** General characteristics of the study cohort.

Variable	Mean (SD^a^)	
Age (years)	60.3 (10.1)	
Time from survey to surgery (days)	11.0 (15.1)	
BMI b (kg/m2)	26.7 (4.80)	
**Variable**		**N (%)**
Active smoker		
Yes		16 (5.1)
No		293 (93.0)
Missing		6 (1.9)
ASA-classification c		
ASA I		124 (39.4)
ASA II-III		191 (60.6)
Type of cancer		
DCIS d		11 (3.5)
Ductal carcinoma		227 (72.1)
LCIS e		2 (0.6)
Lobular carcinoma		37 (11.7)
Other or unclear		38 (12.1)

a Standard deviation.

b Body mass index.

c ASA I normal, healthy patient, ASA II patient with mild systemic disease, ASA III patient with severe systemic disease.

d Ductal carcinoma in situ.

e Lobular carcinoma in situ.

### 3.1 Psychosocial well-being

The mean value for psychosocial well-being was 70.8 (SD 15.0). A total of 313 patients (99.4 %) answered this subscale (Table 2). In regression analyses, higher BMI was associated with a lower score in this scale (R^2^ = 0.049, p = 0.001, β = −0.51, CI 95 % = −0.88 to −0.14, p = 0.007). Age (p = 0.06) or ASA-class (p = 0.19) did not affect the results in this scale (Table 3).

**Table 2:** Preoperative BREAST-Q scores in the present study and previously published normative values for Psychosocial Well-Being, Sexual Well-Being, Satisfaction with Breasts and Physical Well-Being: Chest.

Scale	Number of patients, N (%)	Present study Mean (SD^a^)	Normative values Mean (SD) [7]
Psychosocial Well-Being	313 (99.4)	71 (15)	71 (18)
Sexual Well-Being	292 (92.7)	58 (15)	56 (18)
Satisfaction with Breasts	314 (99.7)	60 (16)	58 (18)
Physical Well-Being: Chest	313 (99.4)	82 (16)	93 (11)

a Standard deviation.

**Table 3:** Linear regression models for Psychosocial Well-Being, Sexual Well-being, Satisfaction with Breasts, and Physical Well-Being: Chest.

Scale	N^c^	R^b^^d^	P-value
	β^e^	CI 95 %^f^	P-value
**Psychosocial Well-Being**	**313**	**0.049**	**0.001**

Age (years)	0.16	−0.007–0.32	0.06
BMI (kg/m2) a	−0.51	−0.88–−0.14	0.007
ASA^b^	−2.48	−6.16–1.20	0.19

**Sexual Well-Being**	**292**	**0.029**	**0.037**

Age (years)	0.02	−0.15–0.20	0.80
BMI (kg/m2)	−0.53	−0.91–−0.14	0.007
ASA	−0.07	−3.93–3.78	0.97

**Satisfaction with Breasts**	**314**	**0.11**	**< 0.001**

Age (years)	−0.04	−0.20–0.13	0.66
BMI (kg/m2)	−1.06	−1.43–−0.68	<0.001
ASA	0.24	−3.48–3.95	0.90

**Physical Well-Being: Chest**	**313**	**0.045**	**0.003**

Age (years)	0.32	0.15–0.49	<0.001
BMI (kg/m2)	−0.16	−0.55–0.22	0.41
ASA	−1.78	−5.64–2.10	0.37

a Body mass index.

b ASA I normal, healthy patient, ASA II patient with mild systemic disease, ASA III patient with severe systemic disease.

c Number of patients.

d R-Squared, coefficient of determination.

e Unstandardized B.

f Confidence interval 95 %.

### 3.2 Sexual well-being

The mean value for sexual well-being was 58.2 (SD 15.1). A total of 292 patients (92.7 %) answered this subscale (Table 2). In regression analyses, higher BMI was associated with a lower score in this scale (R^2^ = 0.029, p = 0.037, β = −0.53, CI 95 % = −0.91 to −0.14, p = 0.007). Age (p = 0.80) or ASA-class (p = 0.97) did not affect the results in this scale (Table 3).

### 3.3 Satisfaction with breasts

The mean value for satisfaction with breasts was 59.9 (SD 15.6). A total of 314 patients (99.7 %) answered this subscale (Table 2). In regression analyses, higher BMI was associated with a lower score in this scale (R^2^ = 0.11, p < 0.001, β = −1.06, CI 95 % = −1.43 to −0.68, p < 0.001). Age (p = 0.66) or ASA-class (p = 0.90) did not affect the results in this scale (Table 3).

### 3.4 Physical well-being: chest

The mean value for the physical well-being of the chest was 81.7 (SD 15.7). A total of 313 patients (99.4 %) answered this subscale (Table 2). In regression analyses, younger age was associated with a lower score in this scale (R^2^ = 0.045, p = 0.003, β = 0.32, CI 95 % = 0.15–0.49, p < 0.001). BMI (p = 0.41) or ASA-class (p = 0.37) did not affect the results in this scale (Table 3).

## 4 Discussion

This study presents preoperative reference values for breast cancer patients using the BREAST-Q. To our knowledge, all previous normative HRQL values have included data derived from women without breast cancer. In particular, no prior studies have reported preoperative normative values in a Finnish population. As a result, the normative values established on healthy women by the original authors of the BREAST-Q are widely used as reference values for evaluating the effect of breast cancer treatments [7]. However, this approach does not consider the long-lasting impact of breast cancer diagnosis on HRQL and psychological well-being [18,19].

This study established reference values for four BREAST-Q scales: Psychosocial Well-being, Sexual Well-being, Satisfaction with Breasts, and Physical Well-being: Chest, included in the BREAST-Q Mastectomy, Breast-Conserving Therapy, and Reconstruction modules [4,6]. All scales apart from the physical well-being of the chest had similar mean values compared to the proposed normative mean scores.

The mean value for psychosocial well-being was 71, in line with the suggested normative mean value for this scale, 71, and within the minimal important difference (MID) estimate for this scale, 4. Sexual well-being (mean 58) was also close to the normative mean value, 56, and within the MID, 4. The mean score for satisfaction with breasts was 60, in line with the normative mean value, 58, and falling within the MID (4) for this scale [20]. Thus, it seems that the breast cancer diagnosis does not directly impact the results in these scales.

In this study, the mean value for the physical well-being of the chest in patients with a recent breast cancer diagnosis was 82, being significantly lower than the normative value, 93, and exceeding the MID, 3, for this scale [20]. This scale measures physical problems in the breast area, including pain, tenderness, and tightness [6]. Early breast cancer is typically either asymptomatic or in case of a presenting symptom, a breast lump without pain. However, the core needle biopsies leading to the cancer diagnosis are generally taken shortly before the first outpatient visit, and the biopsies often induce pain, swelling, tenderness, and hematoma of the breast. Moreover, the breast cancer diagnosis itself may cause mental distress, especially during the time between diagnosis and initial treatment, increasing the experience of breast symptoms [21]. Indeed, it has been suggested that with increased rates of depression and anxiety in breast cancer patients, also the sensation of pain increases, even before treatment [22]. These factors may contribute to the significantly lower physical well-being of the chest in cancer patients compared to the healthy control group. The mean score in this study also differs from previous studies presenting normative values., including healthy women without cancer, suggesting that the awareness of breast cancer alone decreases the physical well-being of the chest, prior to any surgery of the affected area [[8], [9], [10]].

Increases in the body mass index were associated with lower scores of both psychosocial and sexual well-being as well as satisfaction with breasts. It has previously been suggested that higher BMI is associated with lower satisfaction with breasts postoperatively [11,23]. It is also known that BMI negatively affects the general HRQL [24,25]. According to the FinHealth 2017 Study, the mean BMI for women in Finland was 27.7 [26]. In this cohort, the mean BMI was 26.7, being lower than the national mean BMI (p < 0.001) and falling in the overweight range. The present study demonstrates a negative effect of increasing BMI on HRQL, even in the absence of obesity.

Age has been shown to affect HRQL in breast cancer patients [27,28]. In patients treated for breast cancer, younger patients have been suggested to have lower HRQL [29]. In this study, younger patients reported lower physical well-being of the chest. In other scales, age did not affect the HRQL. The mean age at the time of the survey was 60.3 years, ranging from 30 to 84 years. The mean age is in line with the median age for receiving a breast cancer diagnosis [30]. Notably the cohort in prior studies offering normative values for the HRQL, in addition to consisting of women without a history of breast cancer or breast surgery, included mainly women of a younger age [7,8]. This present study provides a valuable advantage by offering more precise reference values that correspond to the age demographics of breast cancer patients.

This study offers baseline HRQL values for patients with diagnosed breast cancer, prior to treatment. We used a breast-specific PROM to evaluate the HRQL of the patients. As such, these results cannot be applied to women without any history of breast cancer but provide an advantage and precision when assessing patients with breast cancer. Although recruiting all patients with recently diagnosed breast cancer at our clinic at the time of the study, the response rate was fairly low, possibly leading to selection bias. No assessment was made of whether the participating women sufficiently represent the patient population as a whole. The relatively low response rate may be attributed, in part, to the emotional distress experienced by the patients having recently been diagnosed with breast cancer and required to absorb a significant amount of information on their first outpatient visit. Although the study cohort remained relatively small, the response rate in this study is above average compared to postal surveys on HRQL [31]. The patients answered the survey on average 11 days before the surgery, giving an authentic assessment of the HRQL. While our study population was smaller than the cohort making up the original normative values, this study is strengthened by including only women with a recent breast cancer diagnosis and no previous breast surgery.

## 5 Conclusions

This present study provides preoperative reference values for the BREAST-Q for women with breast cancer diagnosis. These reference values can be applied to the BREAST-Q modules for Mastectomy, Breast-Conserving Therapy, and Reconstruction. Using these values can help improve the assessment and follow-up of breast cancer patients, allowing a more accurate comparison when evaluating the effect of surgical treatment and postoperative HRQL.

## CRediT authorship contribution statement

**Charlotta Kuhlefelt:** Writing – review & editing, Writing – original draft, Validation, Methodology, Formal analysis, Conceptualization. **Jussi P. Repo:** Writing – review & editing, Validation, Methodology, Conceptualization. **Veera Rasi:** Writing – review & editing, Formal analysis, Data curation. **Tuomo Meretoja:** Writing – review & editing, Validation, Resources. **Tiina Jahkola:** Writing – review & editing, Validation, Resources, Methodology, Conceptualization. **Susanna Kauhanen:** Writing – review & editing, Validation, Supervision, Resources, Methodology, Conceptualization. **Pauliina Homsy:** Writing – review & editing, Validation, Supervision, Methodology, Conceptualization.

## Conflict of interest statements

The authors have no conflicts of interest to declare.

## Ethical approval

This study was conducted in compliance with ethical principles and institutional guidelines. The study protocol was approved by the Helsinki University Hospital ethics committee (HUS/2737/2017, accepted June 12, 2018). Written consent was obtained from all study participants.

## Funding

This work was supported by funding from Helsinki University Hospital Musculoskeletal and Plastic Surgery Research Centre.
